# Friend or foe: assessing the value of animal models for facilitating clinical breakthroughs in complement research

**DOI:** 10.1172/JCI188347

**Published:** 2025-06-16

**Authors:** Felix Poppelaars, V. Michael Holers, Joshua M. Thurman

**Affiliations:** Department of Medicine, University of Colorado School of Medicine, Anschutz Medical Campus, Aurora, Colorado, USA.

## Abstract

Animal experiments have long been a cornerstone of advancements in biomedical research, particularly in developing novel therapeutic strategies for inflammatory and autoimmune diseases. However, these historically important approaches are now facing growing scrutiny for ethical reasons, concerns about translational limitations to human biology, and the rising availability of animal-free research methods. This shift raises a critical question: How relevant and effective are animal models for driving future advancements in today’s research landscape? This Review aims to explore this question within the field of biomedical research on the complement system, critically evaluating the contribution of animal models to the recent advancements and clinical successes of complement-targeted therapies. Specifically, we assess areas where animal studies have been indispensable for elucidating disease mechanisms and conducting preclinical evaluations, alongside instances where findings from animal models failed to translate successfully to human trials. Furthermore, we discuss similarities and differences in the complement system between animals and humans and explore innovations in animal research designed to improve translatability to human biology. By assessing the contributions of animal studies to complement therapeutics, this Review aims to provide insights into animal models’ strengths, limitations, and evolving role in complement research.

## Introduction

The complement system is a network of circulating and membrane-bound proteins (Tables 1, 2, 3, 4, and 5), crucial for immune defense and tissue homeostasis, while driving inflammation and injury during disease (1). As an ancient component of immunity, complement is highly conserved across vertebrates, with primitive forms present even in invertebrates (2). Research on this system, utilizing in vitro assays, animal models, and human samples, has provided key insights into its biology and role in numerous diseases (3). The FDA has currently approved 11 complement inhibitors for 11 disease indications (Supplemental Table 1; supplemental material available online with this article; https://doi.org/10.1172/JCI188347DS1), with more under investigation (4). Although clinical complement inhibition has only recently gained momentum, animal models have demonstrated its therapeutic potential for over 50 years (1, 5). However, the use of animal models is increasingly questioned because of ethical and translational concerns. For anti–neutrophil cytoplasmic antibody–associated vasculitis (AAV), animal models were crucial in uncovering the role of complement in its pathogenesis and achieving approval of the C5a receptor antagonist in this disease (6, 7). In contrast, complement inhibitors for paroxysmal nocturnal hemoglobinuria (PNH) were approved almost entirely based on studies using human blood samples (8, 9). Presently, the clinical use of complement therapeutics enables the direct study of complement inhibition in humans (10). This, combined with innovations in molecular techniques and tools — i.e., large-scale genomics (11, 12), AI-assisted methodology (13, 14), and organoids (15) — raises an important question in complement research: How essential is it to continue conducting animal experiments to achieve future success?

Although quantifying the contribution of animal models to clinical advancements in the complement field is challenging, this Review aims to assess areas where animal studies have made substantial contributions and where they have fallen short or proved unnecessary.

## Animal testing in biomedical research

The rise of animal-free methods, along with concerns about animal rights, ethics, high costs, and translatability, sparked skepticism regarding the continued reliance on animal testing. This shift is reflected in policy changes in the United States (Frank R. Lautenberg Chemical Safety Act — Toxic Substances Control Act) and the European Union (Registration, Evaluation, Authorisation and Restriction of Chemicals Regulation & 223/2009 EU CPR), placing animal research under heightened scrutiny. Additionally, the FDA now permits nonanimal testing alternatives for instances like biosimilar drugs and toxicity (FDA Modernization Act 2.0 — S.5002). Surveys indicate declining public support for animal testing and growing preference for its elimination (16). However, negative media coverage and misinformation contribute to unfavorable perceptions of animal testing (17), while increased awareness of regulations protecting laboratory animals improves attitudes (18). This complicates the determination of whether current negative views stem from misinformation or informed opinions.

Animal testing in research is under strict regulations in North America, the European Union, and several other countries, based on the principles of the 3 Rs (Replacement, Reduction, and Refinement) (summarized in ref. 19). Established over 50 years ago, these principles provide a framework for conducting (more) ethical animal research. In brief, Reduction involves using the minimum number of animals needed for reliable results, while Refinement focuses on minimizing pain and suffering. Replacement entails employing nonanimal methods, when possible, either absolutely or relatively (animals provide organs or tissues for in vitro experiments). Replacement can also involve substituting vertebrates with species that have a reduced capacity to feel pain (i.e., invertebrates or bacteria). Other strategies to reduce animal use include improved study design, method development, and project coordination. In silico (computational modeling), in vitro, and ex vivo approaches can also support the Reduction and Replacement principles.

## Using animals to understand the human complement system

Despite being highly conserved across vertebrates, notable differences still exist between humans and research animals (Tables 1–5) (20). Animal models are selected for their ability to standardize and manipulate, thereby determining causality. Furthermore, research in intact organisms provides context, as the complement system operates in circulation and locally in tissues, while interacting with other systems (1, 3). Animal studies provided valuable insights, but not all findings translate to humans (3, 21). What, then, makes a model suitable for complement research, particularly for developing diagnostics and therapies for human diseases? Besides anatomical, physiological, and disease-related similarities, it is crucial to evaluate aspects of the complement system relevant to the research question, including phylogenetic proximity, sequence alignment, structure, functionality, protein interactions, and expression levels.

### Commonalities in the complement system across species.

**Figure 1:**
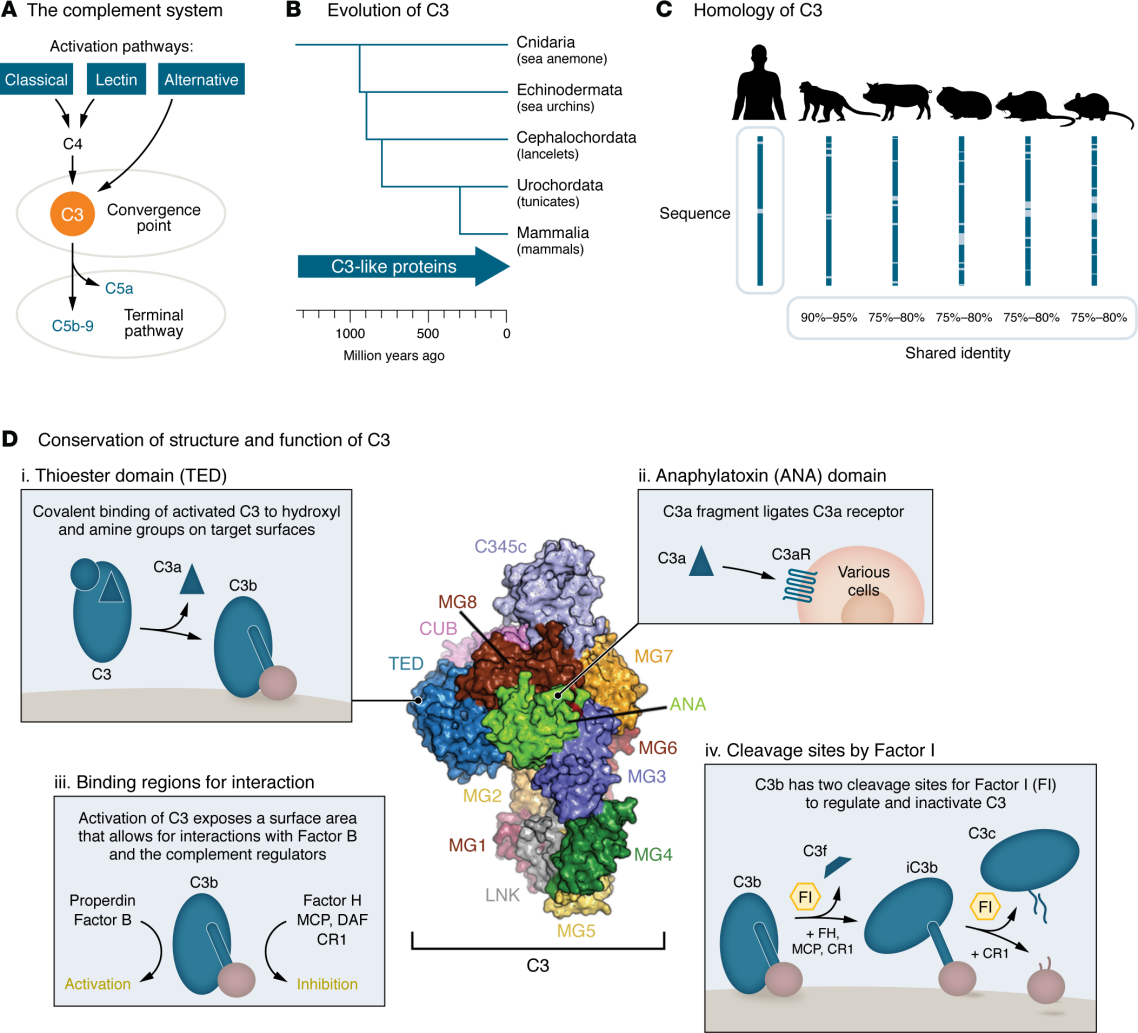
Conservation of structure and function of C3 across species. (**A**) C3 is the central and most abundant circulating complement protein, forming the pivotal convergence point of all pathways. (**B**) Phylogenetic tree illustrating the early emergence of C3-like genes in primitive invertebrates. (**C**) Human C3 protein (UniProt Knowledgebase [UniProtKB]: P01024) exhibits significant homology with other animals, including mouse (UniProtKB: P01027), rat (UniProtKB: P01026), guinea pig (UniProtKB: P12387), pig (UniProtKB: P01025), and cynomolgus monkey (UniProtKB: A0A2K6D5R0). Percentages represent amino acid identity shared with human C3 and were obtained using the Align function on UniProt. (**D**) Human C3 consists of eight macroglobulin domains (MG1–MG8); an ANA domain; a linker (LNK) domain; a C1r/s, Uegf, B (CUB) domain; a TED; and a C345C domain. Structure of human C3 adapted from Zarantonello et al. with permission (368). Moreover, functional characteristics of C3 are conserved even in the most primitive invertebrates: (i) Cleavage of C3 removes the ANA domain (forming C3a) and induces conformational changes (forming C3b), exposing the reactive TED that enables covalent binding to surfaces. (ii) C3a is an ANA that can bind to its receptor (C3aR), leading to pro- and antiinflammatory effects, and is expressed in most species. (iii) Once formed, C3b can interact with Factor B, properdin, and various complement regulators. Factor B binding initiates formation of C3-convertases, which cleaves additional C3 into C3b, thereby creating an amplification loop. Binding of FH, MCP, DAF, and CR1 mediates C3-convertases’ deactivation (via disruption of the C3b–Bb interaction) or degradation (via proteolytic cleavage of C3b). (iv) C3 contains two highly conserved cleavage sites for Factor I (FI), which, in the presence of cofactors such as FH, MCP, or CR1, inhibit further activation and cleave C3. The first cleavage by FI releases C3f, forming inactivated C3b (iC3b). A second cleavage releases C3c from the target-bound C3dg fragment. C3 fragments can still exert functional consequences via interaction with receptors.

Mammals, birds, amphibians, and fish generally possess a complete set of complement genes, with few exceptions (2). C3, a central component of the complement cascade, shows strong conservation across species (Figure 1) (22, 23). Similarly, there is a high degree of interspecies amino acid sequence homology with Factor B, along with collectin-10 and collectin-11 of the lectin pathway (LP) (2, 20, 24). Even though Factor H, a soluble complement regulator, is only 63% identical between humans and mice, the structural organization and functional roles remain highly similar (25). In primitive invertebrates, C3-like molecules retain key structural features analogous to human C3, including the thioester moiety (which enables covalent binding to surfaces), anaphylatoxin domain (C3a fragment), cleavage site (forming C3b), and Factor B binding site (C3-convertase assembly) (22, 23). Furthermore, many complement proteins exhibit functional cross-species reactivity (26). Importantly, protein-protein interactions in the complement system are highly conserved across species, such as C1q with IgG (27), MASPs with mannose-binding lectin (28), Factor H with C3d (25), and CD59 with C8 (29). Reduced homology and loss of cross-reactivity with human counterparts can result from coevolution to preserve key protein interactions, maintaining the fundamental framework of the complement system within species (29). Together, these functional similarities underscore the value of animal models in studying the complement system’s role in human diseases.

Three examples of key paradigm shifts in the understanding of complement biology discovered in animal models and proven relevant to humans will be highlighted. Traditionally, the complement system was regarded as a liver-produced system confined to the circulation. However, research in mice revealed that locally produced complement is crucial for immune responses in diseases. Over 35 years ago, mouse kidneys were found to express and synthesize prominent amounts of complement (30). Later, a series of elegant experiments using a murine kidney transplantation model demonstrated that locally produced C3, rather than circulating C3, is paramount in initiating alloreactivity (31). When wild-type or *C3^–/–^* kidneys were transplanted into *C3^–/–^* or wild-type recipients, wild-type recipients of *C3^–/–^* kidneys exhibited the best outcomes, with 80% graft survival after 100 days. Recently, these observations were verified in humans, where genetic variations in donor C3, Factor B, and Factor H were associated with allograft survival in kidney transplantation, whereas recipient genetics had no effect (32). Another discovery arising from mice is the interaction between the LP and the alternative pathway (AP) via MASP-3, a splice variant of the *MASP1* gene (33). Evidence of MASP-3’s role in AP activation came from *MASP1/3^–/–^* mice, which showed minimal AP activity alongside increased pro-enzyme Factor D levels (34, 35). MASP-1 was found to convert pro-Factor D in vitro (34, 35), but MASP-3 was ultimately uncovered as the main Factor D activator in vivo (36, 37). Findings in humans verified that MASP-3 functions similarly across mammals (38, 39). A final noteworthy example is sex-based differences in the complement system, first reported in *Science* in 1966 (40). Testosterone treatment in sterilized mice enhanced terminal complement activity, whereas estrogen decreased it (40). Recent work verified that female mice have reduced complement activity because of lower levels of terminal components (41). Complement assessments in healthy Norwegian blood donors corroborated that women have lower levels of terminal components and reduced functional activity (42).

### Differences in the complement system among species.

The complement system exhibits notable differences across humans and research animals (Tables 1–5). Even closely related primate species show divergences in complement genetics, circulating levels, and activity (43, 44). Among the pathways, the LP shows the greatest disparities across species (Table 1). In humans and great apes, *MBL2* encodes mannose-binding lectin, while *MBL1* is a pseudogene (45). In contrast, rodents, rabbits, pigs, and rhesus monkeys have two functional genes: *Mbl-a* and *Mbl-c* (45–48). Similarly, while humans and primates possess three ficolins (ficolin-1 to ficolin-3), rodents, rabbits, and pigs have only two (ficolin-A and ficolin-B), with ficolin-3 being a pseudogene (49–51). Additionally, mice exhibit reduced classical pathway (CP) functionality (52). C1r and C1s exist as gene duplicates in mice, whereas humans possess a single gene for each (53, 54). Humans have two C4-encoding genes (*C4A* and *C4B*), while mice have one C4 gene (Table 2), along with a “C4-like” gene for sex-limited protein (Slp) (55). Unlike C4, Slp is exclusively expressed in male mice of certain strains, is not cleaved by C1s, and has low C4 activity (56–58). However, Slp may enhance CP activation by acting synergistically with C4 (58). Furthermore, immunoglobulin (sub)classes differ across species in their ability to activate complement (59).

Functional assays indicate that the AP, compared with other pathways, is relatively more potent in rodents than in humans (60–62). Although Factor H shows high cross-species similarities (Table 3), this does not apply to other members of the Factor H protein family (63). Rodents lack an ortholog of human Factor H-like protein 1 (FHL-1), an alternative splicing variant of the Factor H gene (63). Humans also have Factor H-related proteins (FHR-1 to FHR-5), originating from duplication events of the Factor H gene, leading to structural similarities (64). However, these duplication events occurred after the divergence of rodent and primate lineages (64). Consequently, the structure, domain composition, and sequence of murine FHR genes differ from those in humans (63, 64). The resemblance between FHRs in humans and other animals, regarding distribution and functionality, remains unclear.

Surface regulators and receptors also exhibit major cross-species differences (Tables 4 and 5). Membrane cofactor protein (MCP/CD46) is widely expressed in humans but limited to testes in rodents (65). Furthermore, pigs, bovines, and most primates express MCP on erythrocytes, whereas humans do not (65–68). Although MCP’s cofactor activity for Factor I–mediated cleavage of C3b is conserved across species (66, 69), structural differences exist, as MCP is a receptor for species-specific pathogens (70). Decay-accelerating factor (DAF/CD55) and CD59 are other widely expressed surface regulators in pigs, primates, and humans, preventing complement-mediated cell lysis (71, 72). Mice, however, possess two genes for both regulators, one widely expressed and resembling its human counterpart, and one restricted to the testes (73–75). Remarkably, guinea pigs are the only mammals that lack CD59 (76).

In humans and primates, complement receptor 1 (CR1/CD35) and complement receptor 2 (CR2/CD21) are encoded by separate genes (77), while in rodents, a single gene (*Cr2*) produces both receptors via alternative splicing (78, 79). Additionally, key differences exist in structure, functionality, and expression between rodent and human CR1 and CR2 (77–84). Rodents express another regulator named CR1-related gene/protein Y (*Crry*), which is absent in humans and primates, likely performing regulatory roles of human DAF, MCP, and CR1 (83, 84). The loss of *Crry* in primates is believed to have contributed to the development of a separate CR1 gene (77). The dog genome contains a single *Cr2*-like gene adjacent to two *Crry*-like and two *MCP*-like genes, whereas the gene organization of complement receptors in pigs remains poorly characterized. Finally, although C3a and C5a receptors in humans and mice are functionally similar, their cellular expression shows both overlap and differences (Table 5) (85–87). Among GPCRs, which typically exhibit 85%–98% homology between humans and mice, anaphylatoxin receptors have the lowest homology (61%–65%) (88–90). Single-cell sequencing is enhancing cross-species comparisons of complement mRNA expression in cell types and tissues, revealing both similarities and differences (91, 92).

Species differences in the binding avidities of complement initiators, as well as the composition and potency of complement pathways, can cause divergences in the mechanism of complement activation between animal models and human diseases, despite both being complement mediated (93–95). Although differences in complement across species are often used to critique animal studies, they have also advanced our understanding of human diseases. Interspecies differences and animal studies have been pivotal in identifying MCP as the receptor for measles virus, which infects humans and primates but not rodents (70). MCP is highly homologous between humans and primates, while rodents exhibit key structural and expression differences (96, 97). Experiments with monkey erythrocytes first suggested MCP as a measles receptor. Functional studies with rodent cells provided conclusive evidence that the virus could bind to and infect rodent cells if they expressed human MCP but no native rodent MCP (96, 97). Thus, while differences in the complement system between animals and humans pose challenges for translational research, they can also help uncover human-specific biology.

### Innovations in animal testing for complement research.

Advances in genome engineering have enabled the development of animal models that more accurately mimic aspects of human physiology, enhancing their clinical relevance. Targeted genomic humanization and conditional or inducible gene knockouts in rodents and larger animals have improved biological alignment with humans.

Replacing animal genes with human equivalents has been employed to address interspecies differences and to aid preclinical drug testing of human-specific targets. Identifying human MCP as the measles virus receptor led to the creation of human MCP-transgenic mice, enabling measles infection studies in previously resistant animals (98). Furthermore, since rodents have a single gene (*Cr2*) for CR1 and CR2, *Cr2^–/–^* mice exhibit dual deficiencies. Mice expressing human complement receptors were therefore developed to study their individual roles in vivo (99–101). Similarly, since mice express a single C4 gene (*C4b*), introducing human *C4A* into mice helped uncover the mechanisms underlying the association between *C4A* and schizophrenia in humans (102, 103). However, transgenic expression of human complement has also had unexpected effects. Humanizing C3 in mice triggered C3 glomerulopathy (C3G), a complement-mediated kidney disease, because of impaired regulation of human C3 by mouse inhibitors, causing spontaneous complement activation (104). Alternatively, C3-humanized rats remain healthy and do not exhibit uncontrolled C3 activation (105). Furthermore, humanized Factor H mice normally regulate their AP and attenuate or reverse kidney and eye pathology seen in *Cfh^–/–^* mice (106). Other successful examples of transgenic rodents include knockins of human C1q, C5, C5aR1, C6, DAF, CD59, and C1 inhibitor (107–113).

The development of inducible and/or tissue-specific gene manipulation in mice enables spatial and temporal control in preclinical models. Early applications of this technology involved mice expressing human CD59 on erythrocytes or endothelial cells (114). Subsequent targeting of human CD59 in these mice with a pore-forming toxin created distinct disease models: disseminated intravascular coagulation when endothelial cells expressed CD59 and acute hemolysis when erythrocytes expressed CD59 (114). Tissue-specific knockout mice for properdin identified myeloid cells as the primary source of circulating properdin levels (115), while mice with a conditional deletion of *Crry* in proximal tubular epithelium circumvented the embryonic lethality seen in global knockout mice (116, 117). Overall, tissue- and cell-specific knockouts of complement genes have clarified the distinct functions of local versus systemic complement sources, and their relative significance in infections and inflammatory diseases, sometimes revealing opposing effects (118–121). These models have also uncovered key cell-intrinsic functions of complement (122, 123).

CRISPR/Cas technology has revolutionized genome editing, enabling multiple genetic modifications simultaneously. CRISPR/Cas has created mice with atypical hemolytic uremic syndrome–associated (aHUS-associated) mutations, verifying disease causality and facilitating preclinical drug testing (124, 125). CRISPR/Cas systems also allow for larger gene modifications, such as the humanization of the entire Factor H locus in mice (126). These mice lacked murine Factor H and FHRs but expressed human Factor H along with a normal or mutant FHR-5. The mutant FHR-5, linked to C3G in humans, resulted in a gain of function, causing C3 deposition in the kidney and spontaneous disease (126). Additionally, CRISPR/Cas has generated *Serping1*^–/–^, *C1qa*^–/–^, *Masp3*^–/–^, *Cfd*^–/–^, *Cfhr-e*^–/–^, and *C5*^–/–^ mice (127–132). Traditional genetic modification methods were challenging for large animals; however, CRISPR has enabled the development of *C3*^–/–^ pigs (133). Notably, CRISPR facilitated the creation of pigs with multiple genetic modifications, including human MCP and DAF expression, advancing xenotransplantation toward clinical application (134).

## Evaluating complement-targeted therapies: animal models versus clinical trials

Some diseases have shown drug efficacy in human trials consistent with prior observations in animal models, yet in other cases, clinical trials have failed despite robust animal evidence. It is challenging to discern whether these failures stem from the limitations of animal models or flaws in trial design. Additionally, some anticomplement drugs have been approved based on successful clinical trials in diseases without extensive animal testing, suggesting that animal studies may not always be essential. Conversely, failed trials without strong evidence from animal models raise questions about whether animal studies could have improved the design or predicted failure. Last, although not discussed here, animal testing is crucial for assessing toxicity (discussed in ref. 135).

### Animal studies leading to approved complement inhibitors.

AAV is a group of diseases characterized by vascular inflammation in small vessels. This disease exemplifies how discoveries from animal models can lead to the clinical approval of novel treatments (7). Traditionally, AAV was not considered complement mediated, as circulating C3 and C4 levels are often normal, with minimal tissue deposits of immunoglobulin or complement (136). Clinical trials demonstrated that adding avacopan (a C5aR1 blocker) to existing immunosuppression regimens for maintaining disease remission facilitated faster glucocorticoid tapering, thereby reducing side effects, leading to FDA approval (6). Fifteen years prior, animal studies sparked interest in the complement system in AAV by identifying a critical role for the AP and showing that genetic deletion of Factor B and C5 provided protection (137). Mouse models further revealed the importance of the C5a/C5aR1 interaction in AAV pathophysiology (138). Finally, murine models demonstrated that anti-C5 therapy and blocking C5aR provide protection in AAV (139, 140). Analysis of patients with AAV shows AP activation fragments in blood, urine, and tissues (discussed in ref. 141), verifying findings from animal models. Although human studies may have eventually uncovered the involvement of complement, the initial findings in animal models profoundly accelerated this process.

aHUS is another disease where animal models contributed to and supported the effectiveness of clinical complement inhibition. AP involvement in aHUS was first reported in the 1970s (142–145). Genetic studies associated Factor H mutations, followed by other complement-related gene variants, with the disease (146–150). This suggested that complement activation is central to aHUS, though the precise mechanisms remained unclear. Factor H mutations had also been linked to C3G, which is diagnosed by prominent C3 deposition within the glomeruli (151). Animal models established a causal link between Factor H deficiencies and C3G, as a genetic deficiency in pigs led to spontaneous disease (152). Mice with targeted gene deletions of Factor H verified that complement dysregulation drives C3G (153). A key question remained: Why do some patients with complement dysregulation develop aHUS, while others develop C3G? Factor H mutations in aHUS clustered in the protein’s C-terminus, reducing protection of host cells from unwanted complement activation (154, 155). The seminal study by Pickering et al. revealed that complete Factor H deficiency led to C3G-like disease, whereas mice expressing a Factor H that lacked the last five domains developed aHUS-like disease (156). The structural similarities between Factor H in mice and humans enabled this breakthrough (25), offering the first in vivo evidence that impaired surface recognition by Factor H leads to aHUS. Of note, animal models showed that C5 inhibition in C3G provided only partial protection (157), foreshadowing mixed results in clinical trials with anti-C5 therapy (158). Recent studies suggest that aHUS and C3G probably require different therapeutic approaches to inhibit complement, as loss of Factor D or properdin in mouse models exacerbated C3G but protected against aHUS (125, 159–161).

Myasthenia gravis (MG) is an autoimmune disorder where autoantibodies disrupt neuromuscular transmission. Complement-activating autoantibodies are the primary driver of MG. In 1959, circulating levels of complement were already reported to inversely correlate with the severity of MG symptoms in patients (162). Fifteen years later, evidence emerged that targeting complement could treat MG, with C3 depletion being protective in a rat model (163). In MG patients and animal models, the MAC localizes at the neuromuscular junction (164–166). Animal studies conducted in the late 1980s predicted the success of terminal pathway inhibition in MG, as *C5^–/–^* mice were protected, while anti-C6 Fab antibodies in rats alleviated MG symptoms (167, 168). Overall, animal models have provided compelling evidence for the involvement of complement in MG and its therapeutic potential (169). Although a phase III study of anti-C5 therapy (eculizumab) in refractory MG missed its primary endpoint, positive secondary outcomes showed sustained benefit during the open-label extension phase, leading to FDA approval (170, 171). Later clinical trials with other anti-C5 therapies demonstrated greater improvements in generalized MG (172, 173), resulting in approval of ravulizumab (a long-acting monoclonal antibody against C5) and zilucoplan (a C5-blocking cyclic peptide).

Neuromyelitis optica spectrum disorder (NMOSD) is a relapsing inflammatory disease of the CNS, distinct from multiple sclerosis. Recently, pathogenic autoantibodies targeting the astrocytic water channel aquaporin 4 (AQP4) were identified in most patients with NMOSD, termed AQP4-IgG-seropositive NMOSD (174). In a randomized clinical trial involving patients with AQP4-IgG-seropositive NMOSD, eculizumab reduced the relative relapse risk by 94% compared with placebo (175). Later, ravulizumab demonstrated a similar reduction in relapse risk (176). Animal models of NMOSD were vital in establishing the pathogenic role of anti-AQP4 autoantibodies and the complement system (177–183). Mechanistically, these models uncovered that, in NMOSD, anti-AQP4 autoantibodies bind to astrocytes, triggering complement-mediated cell damage, leading to leukocyte infiltration, cytokine release, and blood-brain barrier disruption (184, 185). This ultimately causes bystander oligodendrocyte injury, myelin loss, and neuronal death (184, 185). Although no publications have reported on anti-C5 therapy in NMOSD models, complement knockouts and complement inhibitors validated the efficacy of complement-targeted therapies in NMOSD (186–188). Overall, the passive transfer of human anti-AQP4 autoantibodies in animal models has been instrumental for uncovering disease pathogenesis and identifying complement as a therapeutic target in NMOSD.

### Unsuccessful clinical trials despite strong animal evidence.

Outcomes in clinical trials of complement-targeted therapies have been most disappointing in ischemia-reperfusion injury (IRI) related to cardiovascular disease and transplantation, along with antibody-mediated transplant rejection. The first large clinical trials of anti-C5 therapy were conducted for cardiac IRI (189). Confidence in targeting complement arose from preclinical studies demonstrating its key role and the efficacy of inhibitors in reducing cardiac IRI in animal models (190, 191). Early studies of a single-chain antibody directed against C5 (pexelizumab) demonstrated promising results in patients with myocardial infarction undergoing reperfusion therapy (192–196). The Assessment of Pexelizumab in Acute Myocardial Infarction trial tested pexelizumab in 5,745 patients with acute myocardial infarction undergoing percutaneous coronary intervention to improve mortality (197). Additionally, the Pexelizumab for Reduction of Infarction and Mortality in Coronary Artery Bypass Graft Surgery–I and –II studies assessed pexelizumab in 3,099 and 4,254 patients receiving cardiac bypass surgery to reduce perioperative myocardial infarction and mortality (189, 198). Together, these trials did not show a consistent significant clinical improvement. Similarly, a soluble form of human CR1 (TP10) was tested in 564 high-risk cardiac surgery patients requiring cardiopulmonary bypass but failed to reduce morbidity or mortality, despite effectively inhibiting complement (199). Previous animal studies showed the inhibitor was highly effective in reducing cardiac IRI (200). Flaws in trial design have been suggested, and post hoc analyses proposed subgroups who might still benefit (201–205). However, it is crucial to recognize that animal models of cardiac IRI fail to accurately replicate the complex clinical setting of patients with myocardial infarction undergoing reperfusion therapy, including comorbidities and concomitant medications (206). For example, the use of heparin, which also affects complement activation, is a treatment aspect not replicated in animal models, potentially confounding results (207).

Although tested in smaller numbers of patients, clinical trials have extensively studied complement inhibitors in solid organ transplantation. Despite these efforts, no evident clinical improvements or regulatory approvals have been achieved to date (208). Clinical trials primarily focused on antibody-mediated rejection (AMR) and IRI and were conducted predominantly in kidney transplantation. Robust data from animal models, including rodents, pigs, and primates, consistently demonstrated the benefit of complement inhibition (209–213). Anti-C5 therapy with eculizumab has been evaluated in nine clinical trials for AMR in transplantation, for either prevention or treatment (ClinicalTrials.gov NCT02013037; NCT01399593; NCT01567085; NCT00670774; NCT01895127; NCT02113891; NCT01095887; NCT01106027; NCT01327573). Similarly, C1 inhibitor has been investigated in six clinical trials for AMR (ClinicalTrials.gov NCT01035593; NCT02936479; NCT02547220; NCT03221842; NCT01147302; NCT01134510). Blocking C5 did not significantly enhance outcomes in highly sensitized patients, nor did it prevent the progression to chronic AMR (214–219). Trials using C1 inhibitor in sensitized recipients also showed underwhelming results (220–222), but therapy may have been underdosed (223). Drugs targeting other complement proteins remain under active investigation in AMR (224). Additionally, complement-targeted drugs have been tested in clinical trials aimed at reducing IRI and improving short-term posttransplant outcomes (225–230), primarily in kidney transplantation. To date, there have been no concrete clinical advancements or regulatory approvals, despite various animal models of IRI predicting clinical success with anti-C5 therapy or C1 inhibitor (231–234).

### Approved complement inhibitors without substantial animal studies.

PNH was the first indication for which eculizumab received FDA approval (8, 9). It was well established that PNH erythrocytes lack CD55 and CD59 because of a genetic mutation affecting their glycosylphosphatidylinositol (GPI) anchor, leading to hemolysis via the insertion of C5b-9 (235). PNH exemplifies FDA approval of complement inhibitors with minimal animal studies. The reasons for limited animal studies in PNH are two-fold: (a) lack of representative animal models (236) and (b) ability to easily collect erythrocytes from affected patients (237), making it straightforward to study complement inhibitors in vitro. Moreover, prior clinical studies had demonstrated sufficient safety of C5 inhibition in humans. Subsequently, studies in patients with PNH treated with eculizumab also advanced our understanding of complement biology and disease mechanisms without further animal use, revealing C3 opsonins on erythrocytes trigger phagocytic uptake in the liver/spleen, causing extravascular hemolysis (10). Cold agglutinin disease (CAD) is another complement-mediated hemolytic anemia that received FDA approval for a complement inhibitor without comprehensive animal model testing. In CAD, autoreactive IgM activates the CP (238). However, since erythrocytes express CD55 and CD59, intravascular hemolysis by C5b-9 is limited and extravascular hemolysis predominates, driven by C3 opsonins (239). Like PNH, no accurate animal models exist, and anticomplement drug efficacy can be evaluated with in vitro assays using patient samples (240, 241). Sutimlimab, a C1s-blocking antibody, prevented opsonization of erythrocytes in vitro and was effective in a phase III study of patients with CAD, leading to FDA approval (241, 242). These results align with studies from the 1960s first suggesting complement’s role in extravascular hemolysis (243).

Age-related macular degeneration (AMD) is a multifactorial eye disease causing retinal degeneration and is the leading cause of blindness in the elderly population. Genetic studies in patients with AMD were the first to uncover the key role of the complement system (63). Nearly two decades ago, three independent studies identified a common genetic variant in Factor H that significantly increased disease risk (244–246). Later research uncovered variants in additional complement genes that contributed to disease risk (12, 247, 248), particularly in the FHRs (63). In 2023, the FDA approved two intravitreal complement inhibitors — pegcetacoplan (C3 inhibitor) and avacincaptad pegol (C5 inhibitor) — as the first treatments for advanced non-neovascular AMD (249–251). Although animal studies have supported and substantiated human genetic findings by confirming the causal role of complement in AMD (106, 252–258), their influence on the approval of complement inhibitors appears limited.

In IgA nephropathy (IgAN), clinical trials led to the approval of complement inhibitors without meaningful animal studies (259). Animal models of IgAN are limited and rarely used to test complement inhibition (260). Decades of observational human data strongly suggested an important role for complement in IgAN. In brief, kidney biopsy data demonstrated that glomerular complement deposition is nearly always present and holds prognostic value (summarized in ref. 261). Extensive biomarker evidence indicated AP activation, including tissue deposition and detection of activation fragments in plasma and urine (261). Unbiased genomics studies linked Factor H and FHR variants to disease risk and activity (13, 261), with circulating levels and renal deposits of FHR also associating with outcomes (63, 261). Collectively, these findings compellingly implicated the AP as a key driver in IgAN. An interim phase III trial analysis showed that iptacopan (Factor B inhibitor) significantly reduced proteinuria in patients with IgAN, leading to accelerated FDA approval (259). This success also highlights how clinical observations can provide a strong rationale for effective clinical trials. Ongoing follow-up will assess iptacopan’s impact on kidney function in IgAN.

A final example is CD55 deficiency with hyperactivation of complement, angiopathic thrombosis, and protein-losing enteropathy (CHAPLE) disease. In 2017, whole-exome sequencing associated loss-of-function variants in the *DAF* gene with early-onset protein-losing enteropathy and thrombosis in 11 individuals with gastrointestinal disorders, subsequently named CHAPLE disease (262). Shortly thereafter, reports demonstrated the efficacy of anti-C5 therapy for this condition (263). Pozelimab, a C5-blocking mAb, resolved clinical and laboratory manifestations of CHAPLE disease in 10 patients during an open-label phase II/III study (264), becoming the only FDA-approved treatment for this condition. *Daf1^–/–^* mice do not exhibit an evident phenotype but are more prone to complement-mediated inflammatory injury (265, 266). As in patients with CHAPLE, *Daf1^–/–^* mice exhibit heightened T cell activity and exacerbated autoimmune-induced colitis (267, 268). The swift approval of a complement inhibitor for CHAPLE disease clearly builds on insights from earlier translational and clinical studies of other complement-mediated diseases. However, given this existing knowledge and the availability of multiple clinical complement inhibitors with well-established safety and efficacy profiles, the necessity of additional animal studies for new indications could be questioned.

## Discussion and remarks

We conclude that animal studies are not the only means of advancing disease understanding or developing complement-targeted therapies, as evidenced by the approval of complement therapeutics with and without reliance on animal studies. Simultaneously, we conclude that animal models remain a valuable tool in the complement field, which currently cannot be replaced. Increasingly available animal-free alternative research methods offer tools that supplement, rather than substitute for, animal-based approaches. Ultimately, every experiment must justify its choice of model — animal or otherwise — since all models are flawed and imperfect. Therapeutic targets supported by human observational genetic evidence are twice as likely to result in approved drugs than targets without human evidence (269). A recent meta-analysis estimated an 86% alignment in positive results between animal models and human studies for therapeutics, yet only 5% progress from animal studies to regulatory approval (270). This suggests that while animal models can accurately predict drug responses in human diseases, their translation is currently limited because of inconsistencies in design between preclinical studies and clinical trials. Therefore, aligning the design of animal studies with clinical trials — by incorporating randomization, blinding, clinically relevant outcomes, and long-term endpoints — could increase the number of treatments that progress from animal studies to regulatory approval. Currently, no data exist on the concordance between positive results from animal-free methods, e.g., organoids, and clinical trial outcomes. Until these methods are proven to be equally effective or superior, they cannot replace animal models.

The clinical efficacy of complement inhibitors is the ultimate validation of its pathophysiological relevance. However, the absence of clinical trials or negative clinical results does not necessarily disprove this. Industry chooses disease indications based on multiple factors, not just animal studies. While membranous nephropathy was among the first kidney diseases in which complement activation was thoroughly documented (271), industry prioritized clinical trials in IgAN. Furthermore, promising complement inhibitors in phase II trials have been discontinued because of shifting business priorities (272). Challenges such as patient recruitment for rare diseases, lengthy study durations, and complex endpoints in chronic conditions further complicate clinical trials. Design flaws may also contribute to unsuccessful outcomes (though this is speculative). For example, LP activation is observed in only one-third of patients with IgAN (261). However, a phase III trial of a MASP-2 inhibitor in IgAN proceeded without assessing LP activation and yielded negative results (NCT03608033). Furthermore, anti-C5 therapy was tested in membranous nephropathy but failed to reduce proteinuria (273). However, the study was prematurely halted, and concerns linger about the therapy being underdosed (274, 275), as proteinuria affects drug pharmacokinetics (276). In rheumatoid arthritis, complement inhibitors have yielded disappointing results so far, but they have been tested only in early-phase trials with few patients and short follow-up periods (277, 278). Consequently, the use of complement inhibitors in these diseases remains an unfinished story.

Animal models, when justified, are invaluable for exploring the complement system in health and disease. We believe this is also evident from our better understanding of disease mechanisms in conditions with approved complement inhibitors, such as aHUS, AAV, and MG, which have animal models, compared with CHAPLE and IgAN, which do not. Additionally, there is a clear need for better diagnostic tools for complement therapeutics, and animal models are extremely useful for developing and validating these tools, such as imaging approaches to detect tissue-bound complement deposits (279). Finally, animal studies have revealed surprising insights into complement’s role in disease, such as the discovery that complement activation can promote tumor growth in animal models (280, 281). While the translation to human disease is tentative, ongoing clinical trials of complement inhibitors for cancer will hopefully answer this question (NCT04919629; NCT04812535). Nevertheless, this research has already expanded our understanding of complement biology (1). In conclusion, when appropriately justified — particularly in relation to translation to human biology and disease — while always considering and addressing ethical concerns, animal models remain a valuable ally to the complement field in the foreseeable future, as they cannot yet be fully replaced.

## Supplementary Material

Supplemental table 1

## Figures and Tables

**Table 5:**
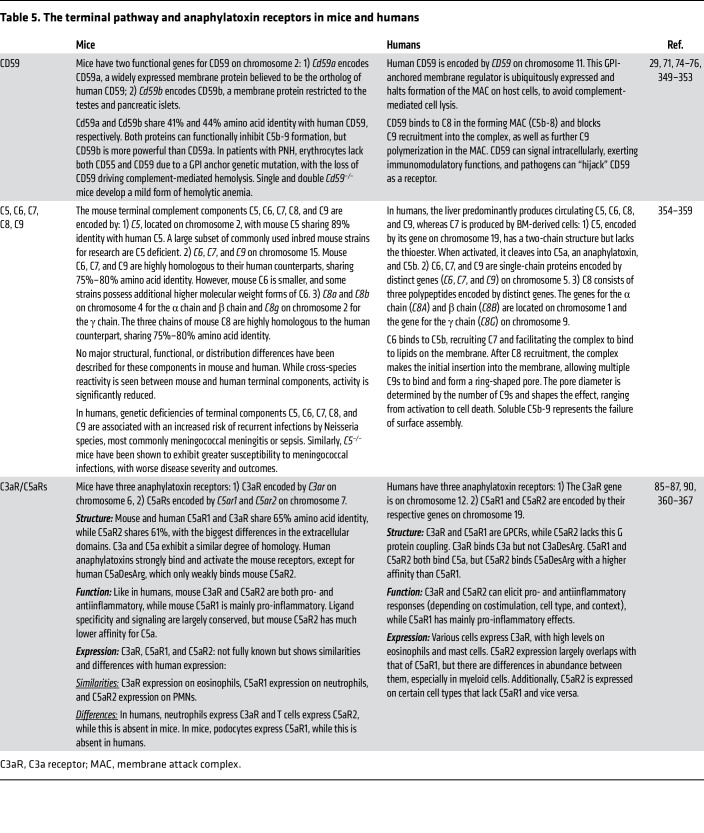
The terminal pathway and anaphylatoxin receptors in mice and humans

**Table 4:**
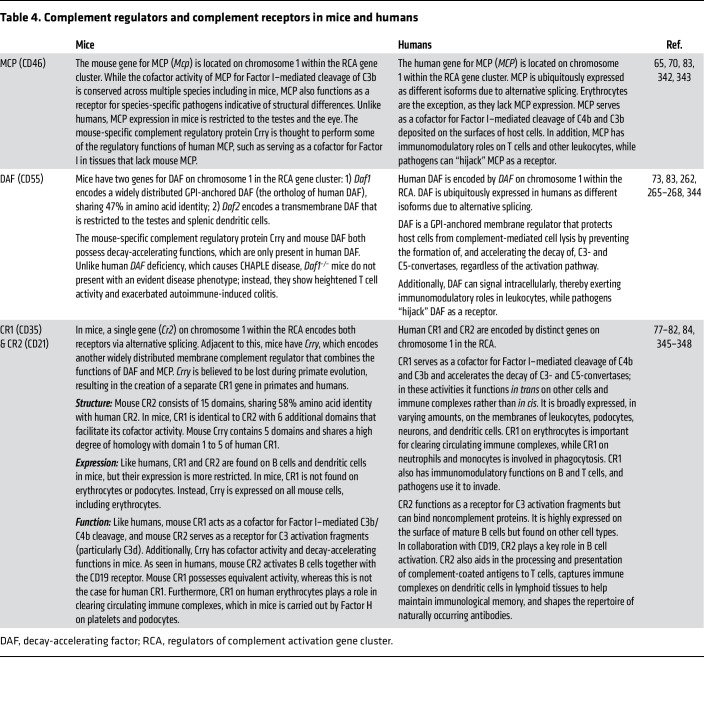
Complement regulators and complement receptors in mice and humans

**Table 3:**
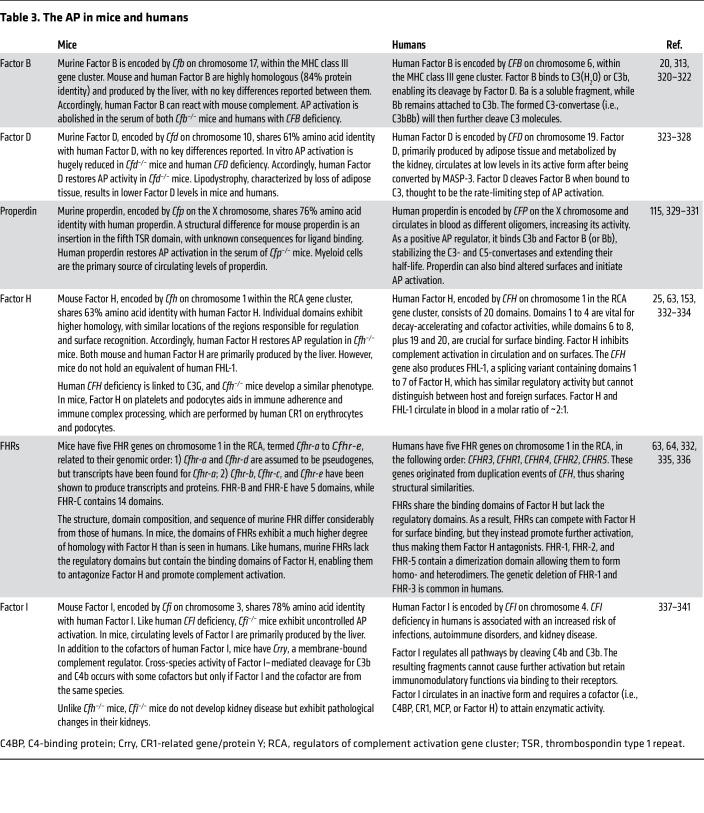
The AP in mice and humans

**Table 2:**
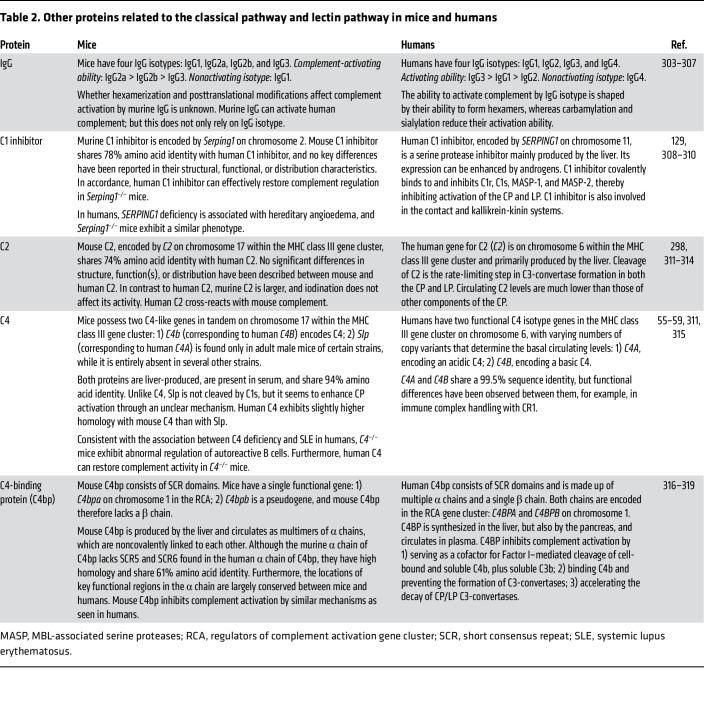
Other proteins related to the classical pathway and lectin pathway in mice and humans

**Table 1:**
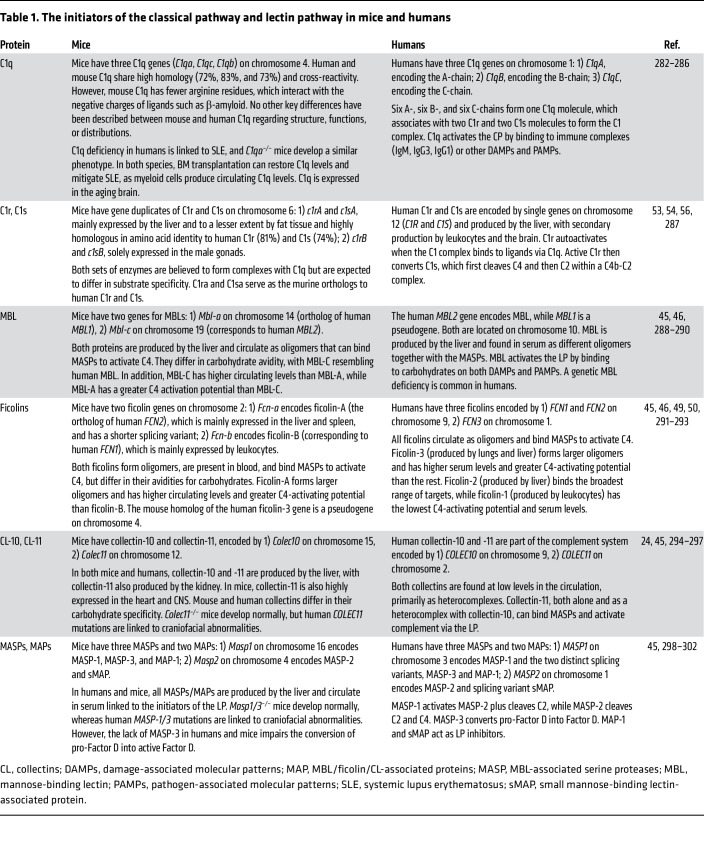
The initiators of the classical pathway and lectin pathway in mice and humans

## Version 1 06/16/2025

Electronic publication
