# Supplementary material for: Friend or foe: assessing the value of animal models for facilitating clinical breakthroughs in complement research
Source: J Clin Invest. 2025 Jun 16;135(12):e188347. doi: 10.1172/JCI188347 (PMC12165806; doi:10.1172/JCI188347)
Supplement: Supplemental table 1 [file jci-135-188347-s109.pdf]

Supplemental information

Table S1: FDA-approved complement-targeted therapeutics as of March 2025

Disease indication	Drug	Target	Type (administration)
Paroxysmal nocturnal haemogobinuria (PNH)	Eculizumab<br>Ravulizumab[1]<br>Bkemv[2]<br>Pegcetacoplan<br>Iptacopan<br>Danicopan[3]	C5<br>C5<br>C5<br>C3<br>Factor B<br>Factor D	mAb (IV)<br>mAb (IV)<br>mAb (IV)<br>small-molecule (SC)<br>small-molecule (PO)<br>small-molecule (PO)
Atypical haemolytic syndrome (aHUS)	Eculizumab<br>Ravulizumab[1]<br>Bkemv[2]	C5<br>C5<br>C5	mAb (IV)<br>mAb (IV)<br>mAb (IV)
Neuromyelitis optica spectrum disorder (NMOSD)	Eculizumab<br>Ravulizumab[1]	C5<br>C5	mAb (IV)<br>mAb (IV)
Generalised myasthenia gravis (gMG)	Eculizumab<br>Ravulizumab[1]<br>Zilucoplan	C5<br>C5<br>C5	mAb (IV)<br>mAb (IV)<br>small-molecule (SC)
Anti-neutrophil cytoplasmic antibody (ANCA)-associated vasculitis (AAV)	Avacopan	C5aR	small-molecule (PO)
Cold agglutinins disease (CAD)	Sutimlimab	C1s	mAb (IV)
Coronavirus disease 2019 (COVID-19)	Vilobelimab[4]	C5a	mAb (IV)
Geographic atrophy (GA) due to age-related macular degeneration (AMD)	Pegcetacoplan<br>Avacincaptad	C3<br>C5	small-molecule (IVI)<br>RNA aptamer (IVI)
CD55 deficiency with hyperactivation of complement, angiopathic thrombosis, and protein-losing enteropathy (CHAPLE) disease.	Pozelimab	C5	mAb (IV)
IgA Nephropathy (IgAN)	Iptacopan	Factor B	small-molecule (PO)
C3 Glomerulopahty (C3G)	Iptacopan	Factor B	small-molecule (PO)

IV, intravenous; *IVI*, intravitreal; *mAb*, monoclonal antibody; *PO*, oral; *SC*, Subcutaneous.

[1] Ravulizumab differs from eculizumab by the substitution of four amino acids, which modifies its pharmacokinetics and pharmacodynamics, extending its half-life to four times that of eculizumab.

[2] Bkemv is an interchangeable biosimilar for Eculizumab.

[3] Danicopan is FDA-approved as an add-on therapy to a C5 inhibitor specifically for treatment-experienced patients with clinically significant extravascular hemolysis.

[4] Vilobelimab received Emergency Use Authorization by the FDA for treating COVID-19 in hospitalized adults when started within 48 hours of invasive mechanical ventilation or extracorporeal membrane oxygenation.
